# Theoretical study of phase stability, crystal and electronic structure of MeMgN_2_ (Me = Ti, Zr, Hf) compounds

**DOI:** 10.1007/s10853-017-1849-0

**Published:** 2017-11-30

**Authors:** M. A. Gharavi, R. Armiento, B. Alling, P. Eklund

**Affiliations:** 1 Thin Film Physics Division, Department of Physics, Chemistry and Biology (IFM), Linköping University, 581 83 Linköping, Sweden; 2 Theory and Modelling Division, Department of Physics, Chemistry and Biology (IFM), Linköping University, 581 83 Linköping, Sweden; 3 Max-Planck-Institut für Eisenforschung GmbH, 40237 Düsseldorf, Germany

## Abstract

Scandium nitride has recently gained interest as a prospective compound for thermoelectric applications due to its high Seebeck coefficient. However, ScN also has a relatively high thermal conductivity, which limits its thermoelectric efficiency and figure of merit (*zT*). These properties motivate a search for other semiconductor materials that share the electronic structure features of ScN, but which have a lower thermal conductivity. Thus, the focus of our study is to predict the existence and stability of such materials among inherently layered equivalent ternaries that incorporate heavier atoms for enhanced phonon scattering and to calculate their thermoelectric properties. Using density functional theory calculations, the phase stability of TiMgN_2_, ZrMgN_2_ and HfMgN_2_ compounds has been calculated. From the computationally predicted phase diagrams for these materials, we conclude that all three compounds are stable in these stoichiometries. The stable compounds may have one of two competing crystal structures: a monoclinic structure (LiUN_2_ prototype) or a trigonal superstructure (NaCrS_2_ prototype; R$\bar{3}$mH). The band structure for the two competing structures for each ternary is also calculated and predicts semiconducting behavior for all three compounds in the NaCrS_2_ crystal structure with an indirect band gap and semiconducting behavior for ZrMgN_2_ and HfMgN_2_ in the monoclinic crystal structure with a direct band gap. Seebeck coefficient and power factors are also predicted, showing that all three compounds in both the NaCrS_2_ and the LiUN_2_ structures have large Seebeck coefficients. The predicted stability of these compounds suggests that they can be synthesized by, e.g., physical vapor deposition.

## Introduction

Thermoelectric materials and devices, which directly convert a thermal gradient into an external voltage, are reliable and low-maintenance power-generating materials used for niche applications such as solid-state cooling or electric power supplying units in deep-space exploration. However, the use of thermoelectrics is presently limited [1] by their low efficiency and high cost. For example, the crustal abundance and global production of tellurium is low [2, 3]. This limits widespread use of the benchmark thermoelectric materials (Bi_2_Te_3_ and PbTe). Thus, there is a need for replacement materials.

The thermoelectric efficiency is directly connected to the dimensionless figure of merit:

$$ zT = \left( {\frac{{S^{2} \sigma }}{\kappa }} \right) \times T, $$

where *S* is the Seebeck coefficient, $\sigma$ is the electrical conductivity, $\kappa$ is the thermal conductivity, and *T* is the absolute temperature [4]. The product $S^{2} \sigma$ is known as the power factor. In the limit of $zT \to \infty$, the Carnot engine efficiency (i.e., the maximum efficiency achievable in a heat engine) is obtained. However, designing materials with higher *zT* values is a difficult challenge, as all three terms are interrelated in a way that typically limits *zT* to below unity in commonly available materials.

In order to overcome this barrier, Slack proposed the phonon glass–electron crystal (PGEC) approach for thermoelectric material design [5–7]: one should seek a material with a high Seebeck coefficient value and engineer it in such a way that it will behave like a crystal for electrons, but scatter phonons similarly to glass. As a result, added material optimization processes are required to increase the *zT* of any given material.

As a starting point for this approach of engineering a high $zT$ material, prior works have suggested cubic scandium nitride (ScN) [8]. The Seebeck coefficient of ScN is relatively large (reaching − 180 $\upmu{\text{V}}/{\text{K}}$ at 800 ${\text{K}}$) and because of its low electrical resistivity, large power factors between 2.5 and 3.5 × 10^−3^ ${\text{Wm}}^{ - 1} {\text{K}}^{ - 2}$ have been reported [9, 10]. Doping and alloying ScN with heavy elements [11, 12] and/or creating artificial layer interfaces such as metal/semiconductor superlattices [13–16] can alter properties and decrease the thermal conductivity, resulting in an enhanced *zT*. Furthermore, ScN can also become *p*-type by Sc-site doping [17, 18]. Although the direction of research is promising, ScN does have a relatively large thermal conductivity [19–22] of approximately 8–12 ${\text{Wm}}^{ - 1} {\text{K}}^{ - 1}$. Scandium and nitrogen are both light atoms compared to their heavier counterparts such as lead, bismuth and tellurium which effectively scatter phonons [23], and artificial interfaces seen in superlattices are synthesized at a sub micrometer scale, while thermoelectric power generation requires millimeter-sized bulk samples [24]. Also, scandium does not have phonon isotope scattering as it is an isotopically pure element.

In a recent paper, Alling [25] addressed these issues by proposing an equivalent ternary based on ScN. Scandium (which is a group-3 element) can be replaced with one group-2 and one group-4 element in a 50/50 proportion to cover the same electron valence. The final compound should then have a MeAEN_2_ stoichiometry, with Me representing a transition metal from the group-4 elements and *AE* belonging to the group-2 (alkaline earth) elements, such as magnesium. TiMgN_2_ was predicted to be stable using density functional theory (DFT). Band structure calculations predicted stoichiometric TiMgN_2_ to have a 1.11 eV band gap using the HSE06 [25, 26] hybrid functional. This methodology has also been used by Tholander et al. [27] to predict zinc-based group-4 transition metal nitride stability and crystal structure. While much research has been done regarding Ti–Si–N [28–30] and Ti–Al–N [31–34] which show superior hardness and/or oxidization resistance compared to TiN, there are much fewer studies reported for Ti–Mg–N [35–39]. TiMgN_2_ may crystallize in the B1–L1_1_ superstructure [25], which could open a new opportunity for hard coating research by inter-layer dissipation of heat or research for hard coatings with better mechanical properties.

In this paper, we continue the work in investigating ternary structures based on ScN. We also computationally study the phase stability, band structure, Seebeck coefficient and power factor of two more candidate compounds potentially useful in thermoelectric applications, ZrMgN_2_ and HfMgN_2_. As Ti, Zr and Hf belong to group 4 of the periodic table, all three share similar physical and chemical properties, and it can be assumed that any stable Ti-based ternary may also exist for Zr and Hf.

## Computational details

Over 60 different and chemically stoichiometric crystal structures registered in the Inorganic Crystal Structure Database (ICSD) [40] were studied in order to calculate the formation enthalpy of Ti–Mg–N, Zr–Mg–N and Hf–Mg–N and prepare the necessary phase diagrams. Although the binary nitrides are well known, TiMgN_2_, ZrMgN_2_ and HfMgN_2_ are not present in either the Materials Project database [41] or the ICSD. Half of these crystal structures follow the *Me*MgN_2_ stoichiometry, while the remaining crystal structures belong to various Mg-, Ti-, Zr- and Hf-based ternaries. In addition, the opposite sequence, Mg*Me*N_2_, was also studied in case some structures would show a different phase when switching the positions of the metal atoms in their respective sublattice.

First-principles calculations were employed using DFT [42, 43] with the projector augmented wave method (PAW) [44] implemented in the Vienna ab initio simulation package (VASP) [45–47] version 5.2. Electronic exchange correlation effects and the electronic band structure were modeled with the generalized gradient approximation (GGA) using Perdew–Burke–Ernzerhof (PBE) functional [48]. It should be noted that the Kohn–Sham gaps of standard GGA calculations are systematically smaller than experimental band gaps, but for the present work this is not an issue since we are mostly concerned with dismissing metallic compositions. To the extent that we identify relevant compounds, they can be further investigated by in-depth theoretical work and/or by laboratory synthesis of the three ternary nitrides. The plane wave energy cutoff was set at 400 eV. The required structure files for the crystal structures were obtained from the ICSD and converted to VASP input files using cif2cell [49]. Phase diagrams were prepared using the software package Pymatgen (Python Materials Genomics) [50], the band structure illustrations by the high-throughput toolkit (httk) [51] and the crystal structures by VESTA [52]. For the phase diagrams, the formation energy per atom was calculated for each ternary compound and related to competing ternary stoichiometries and neighboring binary compounds. The Materials Project database provided the formation enthalpies of all of the binaries (TiN, ZrN, HfN, Mg_3_N_2_, etc.).

The present work uses the same correction of the N_2_ energy as used in the Materials Project, based on work by Wang et al. [53] as standard GGA exchange–correlation functionals in DFT are known to, in general, have systematic errors in the prediction of energy differences between solid and gas phase systems [54]. Hence, to accurately reproduce the formation energy of a system relative to a gas end point, it is common to adjust the gas phase energy.

The calculations used an 11 × 11 × 11 *k*-point mesh for Brillouin zone sampling and were executed with the Monkhorst–Pack scheme [55]. For band structure calculations, the tetrahedron method was used in order to obtain band gap values with spin polarization included [56].

Finally, the Seebeck coefficient *S* and power factor $S^{2} \sigma \tau^{ - 1}$ (being the charge carrier relaxation time) of the predicted semiconductors is calculated at room temperature and 600 K as functions of the chemical potential using Boltzmann transport theory with the constant relaxation time approximation. We use the software BoltzTraP [57] on DFT calculations with a 40 × 40 × 40 *k*-point mesh for Brillouin zone sampling.

## Results

### TiMgN_2_

**Figure 1:**
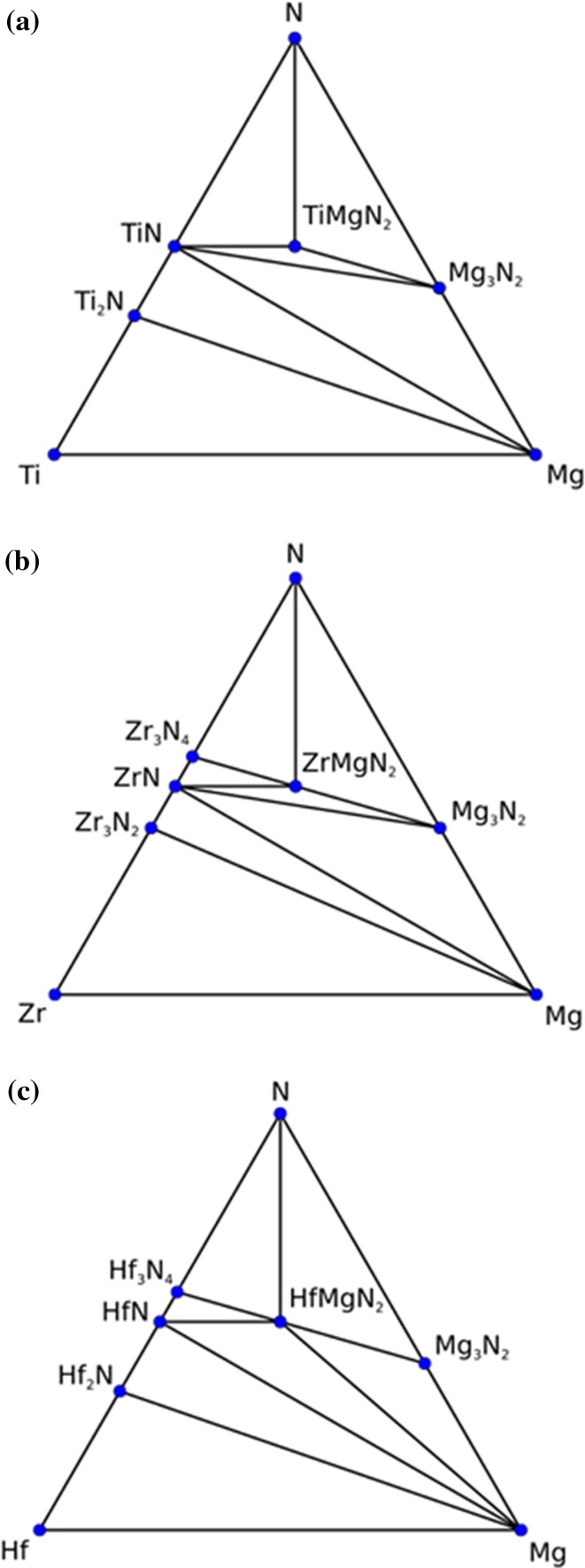
Phase diagram for **a** Ti–Mg–N, **b** Zr–Mg–N and **c** Hf–Mg–N. Only predicted stable structures located on the convex hull are shown. Only the *Me*MgN_2_ stoichiometry is predicted to be stable

Figure 1a shows the phase diagram for Ti–Mg–N. Although 28 different crystal structures other than those that follow the *Me*MgN_2_ formula (such as Ca_4_TiN_4_ [58], perovskite CaTiO_3_ [59], Ti_2_AlN and Ti_4_AlN_3_ MAX-phases [60]) were tested, only the ordered TiMgN_2_ stoichiometry is found to be thermodynamically stable relative to known and investigated phases with the other ordered stoichiometries being either unstable or metastable. Random Ti_1−*x*_Mg_*x*_N solid solutions with the rocksalt structure have, however, been found to be thermodynamically stable for a range of compositions [25]. This precise stoichiometry occurred in 29 of the investigated crystal structures. These include the trigonal NaCrS_2_ (${\text{R}}\bar{3}{\text{mH}}$) superstructure [61], the tetragonal BaNiS_2_ (${\text{P}}4/{\text{n m m Z}}$) superstructure [62], tetragonal LiUN_2_ [63], ZnGeN_2_ [64] (based on the NaFeO_2_-beta structure) and the inverse-MAX BaCeN_2_ [65].

In order to differentiate between these structures, Table 1 lists a selected group of examples with their respective formation enthalpies. These results show that crystallization into the NaCrS_2_ is the most likely outcome with a − 1.299 eV formation enthalpy and a 0.04 eV difference compared to the LiUN_2_ structure which agrees with the findings mentioned in Ref. [25]. It should be noted that the difference between the formation enthalpies of these two crystal structures would most likely mean that NaCrS_2_ is the preferred structure, but LiUN_2_ is also studied for any comparison needed between TiMgN_2_, ZrMgN_2_ and HfMgN_2_.

**Table 1:** Formation enthalpies for TiMgN_2_ crystallized in five different structures

TiMgN_2_		
ICSD id	Formation enthalpy (eV/atom)	Nitride example
82537	− 1.299	SrZrN_2_ (NaCrS_2_)
98663	− 1.260	LiUN_2_
74904	− 1.005	BaZrN_2_ (BaNiS_2_)
15144	− 1.234	ZnGeN_2_ (NaFeO_2_-beta)
74791	− 1.222	BaCeN_2_, inverse-MAX

The ICSD id is the entry identification number which the structure is based on. The nitride examples represent actual existing ternary compounds and the respective structures which they crystallize in

**Figure 2:**
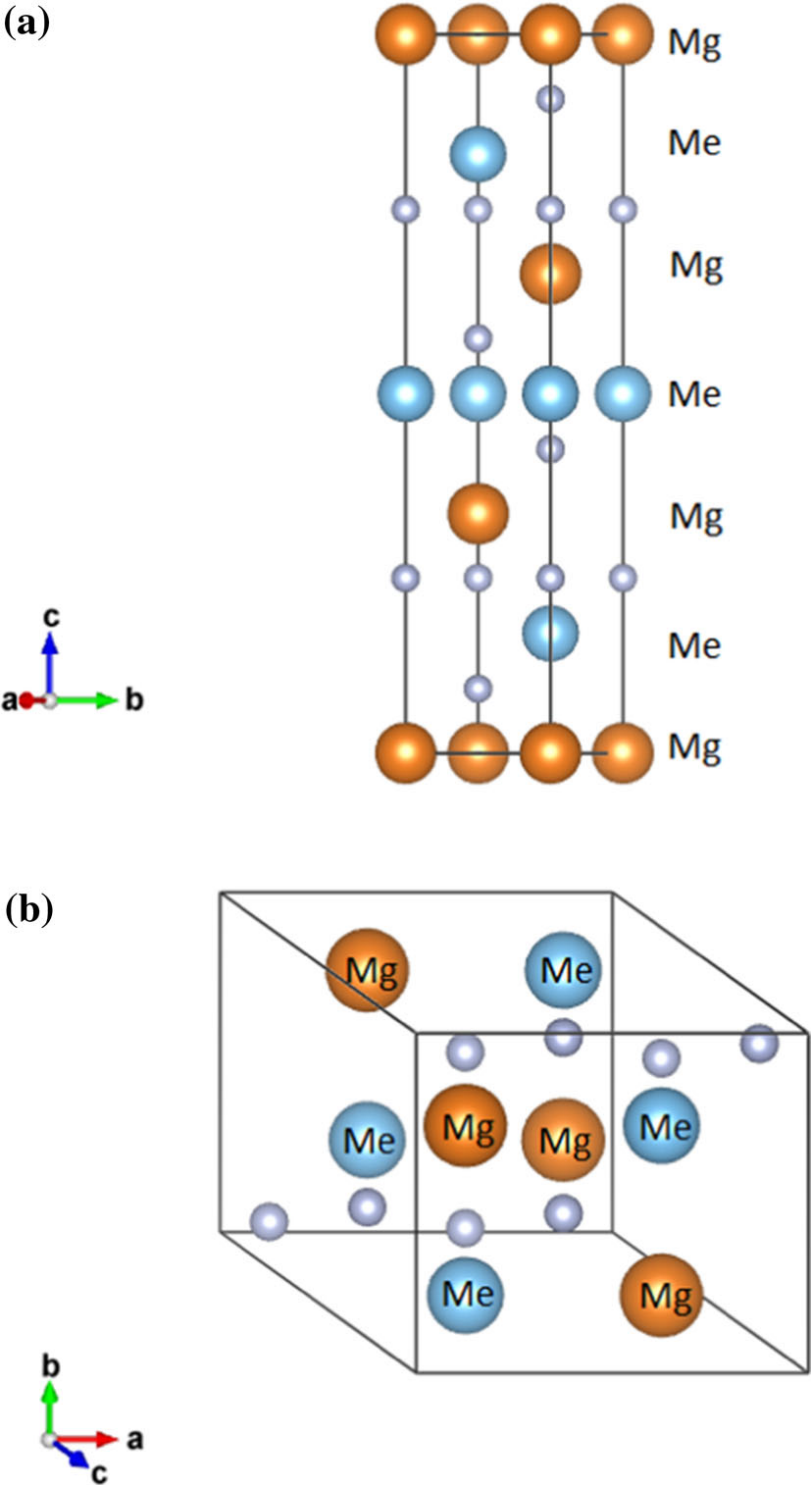
3D visualization of *Me*MgN_2_ crystallized into: **a** the NaCrS_2_ (trigonal unit cell) and **b** the LiUN_2_ (relaxed monoclinic unit cell) structure

**Figure 3:**
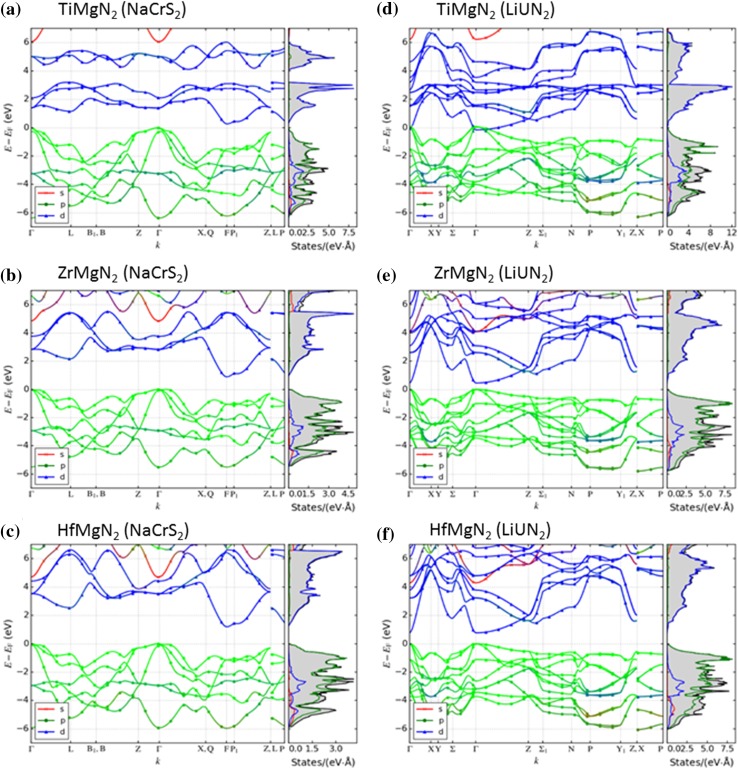
Band structure of **a** TiMgN_2_, **b** ZrMgN_2_ and **c** HfMgN_2_ for the NaCrS_2_ structure (left column) predicting an indirect band gap for all three compounds. Band structure of **d** TiMgN_2_, **e** ZrMgN_2_ and **f** HfMgN_2_ for the LiUN_2_ structure (right column) predicting a direct band gap for ZrMgN_2_ and HfMgN_2_. Note: s, p and d indicate the relative contributions to the total sum, s + p + d, not the absolute projected values

Both crystal structures are shown in Fig. 2. The results suggest that TiMgN_2_ will crystallize into the NaCrS_2_ superstructure (also viewed as a NaCl-B1 superstructure that includes three alternating layers of Ti and Mg) which could cause phonon scattering at the interface of each layer as mentioned in the introduction. Figure 3a, d shows the band structures for TiMgN_2_ in the NaCrS_2_ and LiUN_2_ structures. According to these results, TiMgN_2_ is a semiconductor with a Kohn–Sham PBE band gap of 0.26 eV in the NaCrS_2_ structure (Fig. 3a). However, the case for LiUN_2_ (Fig. 3d) is different, as band structure calculations show no band gap, i.e., predicting metallic properties. It is possible that TiMgN_2_ could crystallize in the LiUN_2_ structure as a metastable phase. Table 2 shows the lattice parameters and the band gap energy in both crystal structures. These results show that although the trigonal NaCrS_2_ crystal structure remains with only the lattice parameters changing, the LiUN_2_ structure relaxes from tetragonal to monoclinic according to the calculated unit cell lattice parameters.

**Table 2:** Lattice parameters, unit cell volumes and band gap values for TiMgN_2_ crystallized in both NaCrS_2_ and LiUN_2_

Crystal structure	Compound (*Me*MgN_2_)	*a* (Å)	*b* (Å)	*c* (Å)	Volume (Å^3^)	Band gap (eV)
NaCrS_2_ (trigonal)	TiMgN_2_	2.9997 $\alpha = 90^\circ$	2.9997 $\beta = 90^\circ$	14.8838 $\gamma = 120^\circ$	115.9849	Indirect: 0.26
LiUN_2_ (tetragonal relaxed into monoclinic)	TiMgN_2_	5.9777 $\alpha = 90^\circ$	5.9777 $\beta =$ $55.1530^\circ$	5.2309 $\gamma = 90^\circ$	153.3962	No gap: metallic?

**Figure 4:**
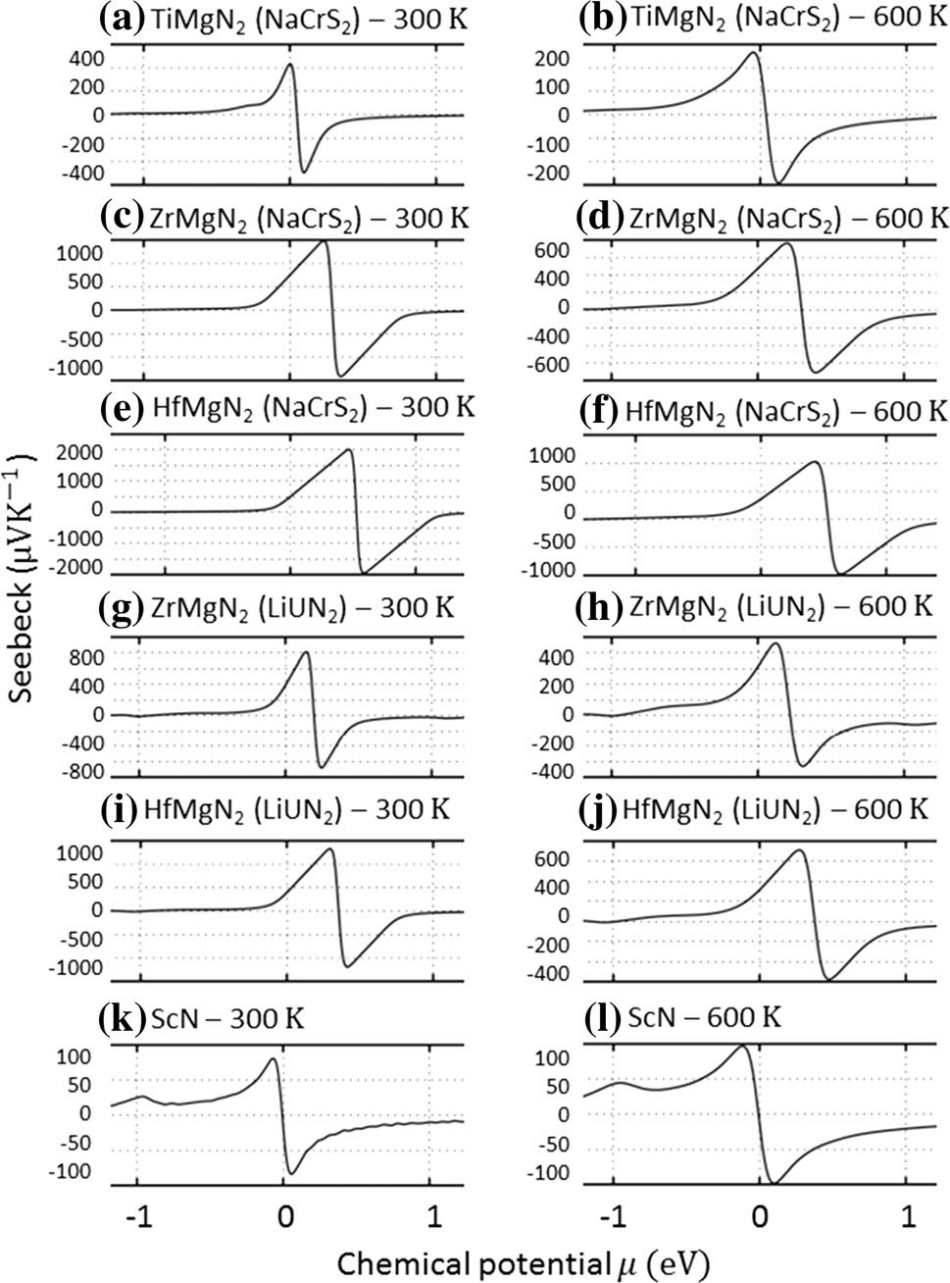
Seebeck coefficient value versus chemical potential of **a** TiMgN_2_, **b** ZrMgN_2_ and **c** HfMgN_2_ for the NaCrS_2_ and LiUN_2_ structures at room temperature and 600 K. The Seebeck coefficient value of ScN is also added for comparison

**Figure 5:**
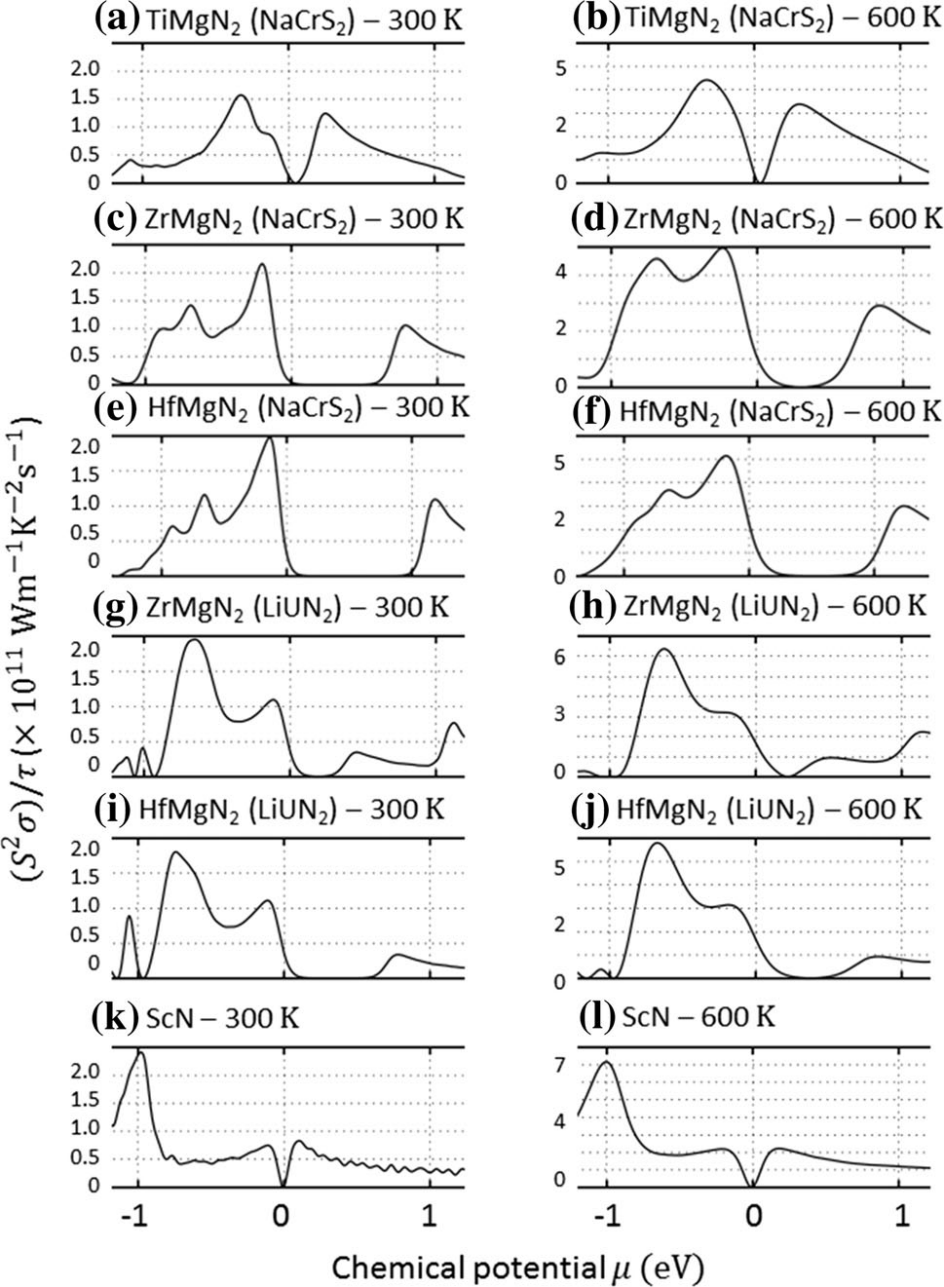
$\left( {S^{2} \sigma } \right)/\tau$ value versus chemical potential of **a** TiMgN_2_, **b** ZrMgN_2_ and **c** HfMgN_2_ for the NaCrS_2_ and LiUN_2_ structures at room temperature and 600 K. The $\left( {S^{2} \sigma } \right)/\tau$ value of ScN is also added for comparison

Figure 4a, b shows the Seebeck coefficient of TiMgN_2_ versus the chemical potential at room temperature and 600 K, respectively. Only the NaCrS_2_ structure was studied as the LiUN_2_ structure was predicted with no band gap. These results show relatively high Seebeck coefficient values at the Fermi level. Figure 5a, b shows $\left( {S^{2} \sigma } \right)/\tau$ versus the chemical potential at room temperature and 600 K, respectively. Depending on the assumed relaxation time, predicted power factor values could exceed those of ScN (Fig. 5k, l).

### ZrMgN_2_

Figure 1b shows the phase diagram for ZrMgN_2_. Also here the only stable ternary has the *Me*MgN_2_ stoichiometry. As for the preferred crystal structure, formation enthalpies for the selected crystal structures are shown in Table 3. In contrast to TiMgN_2_, the LiUN_2_ structure competes with the NaCrS_2_ structure with less than 0.01 eV formation enthalpy difference.

**Table 3:** Formation enthalpies for ZrMgN_2_ crystallized in five different structures

ZrMgN_2_		
ICSD id	Formation enthalpy (eV/atom)	Nitride example
82537	− 1.307	SrZrN_2_ (NaCrS_2_)
98663	− 1.311	LiUN_2_
74904	− 0.897	BaZrN_2_ (BaNiS_2_)
15144	− 1.230	ZnGeN_2_ (NaFeO_2_-beta)
74791	− 1.223	BaCeN_2_, inverse-MAX

The predicted band structures are shown in Fig. 3b, e. In both cases, ZrMgN_2_ is a semiconductor regardless of crystal structure. However, for the NaCrS_2_ crystal structure we find an indirect Kohn–Sham PBE band gap of 0.89 eV and for the LiUN_2_ structure, a direct band gap of 0.46 eV. The respective lattice parameters and band gap energy are shown in Table 4. ZrMgN_2_ relaxes in a similar way as TiMgN_2_ with the NaCrS_2_ structure remaining the same while the tetragonal LiUN_2_ structure relaxes into a monoclinic structure according to the calculated unit cell lattice parameters.

**Table 4:** Lattice parameters, unit cell volumes and band gap values for ZrMgN_2_ crystallized in both NaCrS_2_ and LiUN_2_

Crystal structure	Compound (*Me*MgN_2_)	*a* (Å)	*b* (Å)	*c* (Å)	Volume (Å^3^)	Band gap (eV)
NaCrS_2_ (trigonal)	ZrMgN_2_	3.2077 $\alpha = 90^\circ$	3.2077 $\beta = 90^\circ$	15.3237 $\gamma = 120^\circ$	136.5495	Indirect: 0.89
LiUN_2_ (tetragonal relaxed into monoclinic)	ZrMgN_2_	6.3113 $\alpha = 90^\circ$	6.3113 $\beta = 55.3470^\circ$	5.5498 $\gamma = 90^\circ$	181.8447	Direct: 0.46

Figure 4b, g (room-temperature calculations) and Fig. 4d, h (600 K calculations) shows the Seebeck coefficient of ZrMgN_2_ versus the chemical potential in the NaCrS_2_ and the LiUN_2_ structures. These results show an increase in the Seebeck coefficient values and a slight shift in the chemical potential compared to TiMgN_2_ with higher values seen in the NaCrS_2_ structure. Figure 5b, g and d, h shows the $\left( {S^{2} \sigma } \right)/\tau$ versus chemical potential at room temperature and 600 K, respectively. These results predict power factor values close to the Fermi level which are larger than those of ScN (Fig. 5k, l).

### HfMgN_2_

Figure 1c shows the phase diagram for Hf–Mg–N. Similar to both TiMgN_2_ and ZrHfN_2_, the HfMgN_2_ stoichiometry is predicted to be stable. Table 5 compares a selected group of crystal structures and shows the NaCrS_2_ and LiUN_2_ structures with similar formation enthalpies (less than 0.01 eV difference), thus predicting a competition between the two structures.

**Table 5:** Formation enthalpies for HfMgN_2_ crystallized in five different structures

HfMgN_2_		
ICSD id	Formation enthalpy (eV/atom)	Nitride example
82537	− 1.447	SrZrN_2_ (NaCrS_2_)
98663	− 1.453	LiUN_2_
74904	− 1.034	BaZrN_2_ (BaNiS_2_)
15144	− 1.360	ZnGeN_2_ (NaFeO_2_-beta)
74791	− 1.362	BaCeN_2_, inverse-MAX

Figure 3c, f shows the predicted band structures for both NaCrS_2_ and LiUN_2_. Similar to ZrMgN_2_, an indirect band gap of 1.19 eV is predicted for the NaCrS_2_ structure, while a 0.77 eV direct band gap is predicted for the LiUN_2_ structure. The respective lattice parameters and band gap energies are shown in Table 6. Similar to TiMgN_2_ and ZrMgN_2_, HfMgN_2_ preserves the trigonal NaCrS_2_ structure but relaxes from tetragonal LiUN_2_ into a monoclinic structure.

**Table 6:** Lattice parameters, unit cell volumes and band gap values for HfMgN_2_ crystallized in both NaCrS_2_ and LiUN_2_

Crystal structure	Compound (*Me*MgN_2_)	*a* (Å)	*b* (Å)	*c* (Å)	Volume (Å^3^)	Band gap (eV)
NaCrS_2_ (trigonal)	HfMgN_2_	3.1679 $\alpha = 90^\circ$	3.1679 $\beta = 90^\circ$	15.2463 $\gamma = 120^\circ$	132.5114	Indirect: 1.19
LiUN_2_ (tetragonal relaxed into monoclinic)	HfMgN_2_	6.2300 $\alpha = 90^\circ$	6.2300 $\beta = 55.5036^\circ$	5.5001 $\gamma = 90^\circ$	175.9362	Direct: 0.77

Figure 4e, i (room-temperature calculations) and Fig. 4f, j (600 K calculations) shows the Seebeck coefficient of HfMgN_2_ versus the chemical potential in the NaCrS_2_ and the LiUN_2_ structures. These results show an increase in the Seebeck coefficient values and a larger shift in the chemical potential compared to both TiMgN_2_ and ZrMgN_2_ with higher values seen in the NaCrS_2_ structure. Figure 5e, i and f, j shows the $\left( {S^{2} \sigma } \right)/\tau$ versus chemical potential at room temperature and 600 K, respectively. These results predict power factor values almost equal to those of ZrMgN_2_ and larger than that of ScN close to the Fermi level (Fig. 5k, l).

## Discussion

For ZrMgN_2_ and HfMgN_2_, the formation enthalpies of the NaCrS_2_ and the LiUN_2_ structure are close, within the accuracy of our approach. This suggests that both of these structures may be possible to synthesize, i.e., with the one higher in energy as a long-lasting metastable state. The shifting between the NaCrS_2_ and the LiUN_2_ structures could be done by choosing suitable substrates for epitaxial stabilization during the synthesis process. Despite that we cannot with certainty determine which of the structures for ZrMgN_2_ and HfMgN_2_ are thermodynamically stable, both are semiconductors. This motivates future studies on synthesis for thermoelectrics and other applications. It should be noted that the NaCrS_2_ structures show indirect band gaps with larger values and large slopes for the density of states at the Fermi level compared to their direct band gap counterparts in the LiUN_2_ structure. Another feature seen in all three compounds is the relation between band gap values and lattice parameters with the transition metal, *Me*. As the smaller Ti atom is replaced with the larger Zr atom, the lattice parameters, cell volume and band gap value increase, which is expected. However, only the band gap value increases when Zr is replaced with Hf as the *f* orbital electrons are not effective at screening the increasing charge, resulting in similar atomic size (lanthanide contraction [66]) and similar lattice parameters.

Although the present results are promising, actual attempts to synthesize these prospective compounds would be important. Similar to the synthesis of MAX-phase [67] thin films, it should be possible to synthesize ordered TiMgN_2_, ZrMgN_2_ and HfMgN_2_ outside thermodynamic equilibrium in a magnetron sputtering system. All of the mentioned elements are vacuum compatible, and one could use the deposition parameters needed for stoichiometric TiN, ZrN, HfN and Mg_3_N_2_ to reach a ${\text{Me}}/{\text{Mg }} = 1$ ratio and fine-tune the *Me*MgN_2_ stoichiometry. References [36, 38] note the deposition temperature for rocksalt (Ti, Mg)N alloys to be between 200 and 300 °C with oxidization resistance close to 700 °C (suitable for mid-temperature thermoelectric applications). If the layered NaCrS_2_ superstructure is preferred, it would be advisable to use either high-temperature direct growth or low-temperature deposition, followed by high-temperature annealing [68] (in ammonia or nitrogen). In this case, GaN or SiC [69] substrates could be considered for their suitable lattice constant and thermal stability.

As for the thermoelectric properties, the calculated Seebeck coefficient values show that in the range of a moderate change in the Fermi level, high room-temperature Seebeck coefficient values can be achieved (Fig. 4), although it seems that HfMgN_2_ is either an insulator or would require elemental doping due to the larger shift in the chemical potential.

Note that what we have calculated is the power factor divided by the relaxation time. The results (Fig. 5) can be used as an estimate of the difference in thermoelectric performance at various doping levels between the studied compounds and known materials, e.g., ScN, as shown for comparison in Figs. 4k, l and 5k, l. However, such a comparison is made under the assumptions that the constant relaxation time approximation holds sufficiently well and that the relaxation time for the compounds is similar. For more precise predictions, the relaxation time value needs to be obtained from experimental data, as it can for example for common thermoelectric materials such as Bi_2_Te_3_ [70, 71].

As ordered TiMgN_2_, ZrMgN_2_ and HfMgN_2_ have not yet been studied experimentally, such data do not exist, and obtaining meaningful numbers for the electrical conductivity is difficult. However, using experimental data from Burmistrova et al. [19] and the classical equation for conductivity ($\sigma = n{\text{e}}^{2} \tau m^{ - 1}$), the constant relaxation time $\tau$ for ScN (which the ternaries were modeled after) is estimated to be equal to $6.5 \times 10^{ - 14}  {\text{ s}}$.

## Conclusions

Theoretical methods were used to study the phase stability and band structure of TiMgN_2_, ZrMgN_2_ and HfMgN_2_. In all three cases, only *Me*MgN_2_ is predicted to be the stable stoichiometry. It is shown that stoichiometric TiMgN_2_ crystallizes into the hexagonal NaCrS_2_ superstructure with a 0.26 eV indirect Kohn–Sham PBE band gap. ZrMgN_2_ and HfMgN_2_ were also studied, which shows tendency to crystallize in both the NaCrS_2_ superstructure and the LiUN_2_ prototype monoclinic structure. Both show semiconducting properties regardless of the crystal structure. ZrMgN_2_ shows a 0.89 eV indirect band gap when crystallizing into the NaCrS_2_ structure, while as crystallization into the LiUN_2_ structure results in a 0.46 eV direct band gap. As for HfMgN_2_, the band gap increases as crystallization into NaCrS_2_ results in a 1.19 eV indirect band gap and crystallization into LiUN_2_ results in a 0.77 eV direct band gap. Lattice parameters and cell volumes increase with the substitution of Ti with Zr, but slightly decrease when Zr is substituted with Hf.

Finally, the Seebeck coefficient and power factor was calculated for all of the semiconducting compounds. The results show that in the range of a moderate change in the Fermi level, high room-temperature Seebeck coefficient values can be achieved.

Thus, the predicted stability and semiconducting properties of these compounds can be further studied both theoretically and experimentally for any prospective thermoelectric properties.
